# 

**Published:** 1896-01

**Authors:** 


### INDEX

TO THE

### HOMŒOPATHIC PHYSICIAN.

### VOLUME XVI, 1896.

PAGE

Abdomen, Fœtus in the ... 293
Advertising Axioms ... 59, 300
Ag[illegible]us Castus. C. L. Olds, M. D. ... 207
Ailanthus-Gland. C. L. Olds, M. D. ... 501
Allen, Dr. J. H ... 59, 540
Allen, J. H., M. D. Bursa-Pastoris ... 342
  Psorinum ... 324
  The Truth Shall Make us Free, 18, 162, 351
Allopathic Progress. Edmund J. Lee, M. D. ... 64
Alumni Association of the Hahnemann College ... 204
Amalgam and Kindred Poisons. Henry Sheffield, M. D. ... 228
American Homœopathist ... 249
American Institute of Homœopathy, 98, 251, 537
American Medical Association, The ... 204
Ammonium-bromatum in Bronchial Troubles. Dr. H. Goullon ... 244
Annales D'Oculistique. Review of ... 56
Annales D'Oculistique ... 453
Annales D'Oculistique. George T. Stevens, M. D. ... 491
Annual Meeting Society of Homœopathicians ... 249
Antimonium Tartaricum. Clinton Enos, M. D. ... 22
Anti-toxine. Note on ... 49, 239
Anti-toxin. Diphtheria and ... 41
Appendicitis. Its Surgical Treatment. Howard Crutcher, M. D. ... 505
Appendicitis. A Treatise on. By John B. Deaver, M. D. ... 57, 416
Application of the Materia Medica, The. C. M. Boger, M. D. ... 33
Archives of Pediatrics, The. Review of ... 53
Arsenicum-album. Walter M. James, M. D. ... 1, 61, 109
Asthma from a Suppressed Eruption. Dr. Dahlke ... 234

Bacillinum. A Correction. B. L. B. Baylies, M. D ... 407
Banquet, Annual ... 203
Baylies, Dr. B. L. B. ... 299
Baylies, B. L. B., M. D. Bacillinum. A Correction ... 407
  Repetition of the Dose ... 440
Berridge, E. W., M. D. The Great Desideratum ... 449
Better by Wrapping, Yet Worse by Heat. H. C. Morrow, M. D. ... 189
Boardman, E. W., M. D. A Study of Fever Remedies ... 329
Boger, C. M., M. D. The Application of the Materia Medica ... 33
  Cured Symptoms ... 371, 471
  Headache ... 244
  Vertigo Worse from Motion ... 295
Book Notices...50, 102, 153, 195, 245, 295, 336, 372, 416, 452, 539
Brooklyn Hahnemannian Union, Proceedings of ... 47, 267, 408, 445, 532
Burnett's Cod Liver Oil ... 251
Bursa-Pastoris. J. H. Allen, M. D. ... 342

Carleton, Dr. Edmund. ... 251
Cases. Dr. Kunkel, of Kiel ... 43
Cause, The. Amelia A. Waterhouse, M. D. ... 39
Checking List Case Book of Dr. Maro F. Underwood, The ... 247, 372
Children's Homœopathic Hospital ... 156
Chionanthus in Jaundice. H. C. Morrow, M. D ... 271
Chionanthus-virginica. H. C. Morrow, M. D ... 273
Chronic Diseases, Their Peculiar Nature, and Their Homœopathic Cure, The. By Dr. Samuel Hahnemann ... 195
Circumcision Not a Minor Operation. Howard Crutcher ... 235
Close, Stuart, M. D. Imagination in Medicine ... 38
Color-Vision and Color-Blindness. By J. Ellis Jennings, M. D ... 54, 197
Comments on Syphilinum. H. C. Morrow, M. D ... 211
Commercial Travelers' Fair ... 490
Comparison Between the Two Schools, A ... 445
Compend of the Principles of Homœopathy. By Wm. Bœricke, M. D. Review of ... 489
Complementary Remedies. C. L. Olds. 290
Conditions Affected by Storms. C. Carleton Smith, M. D ... 101
Confirmation of Morphine Symptoms. Thos. Skinner, M. D ... 123
Confirmation of Morphine Symptoms. See "Vertigo Worse from Motion" ... 295
Conquest, The. C. F. Menninger, M. D., 304
Cooke, Dr. Mary A ... 155
Crutcher, Howard, M. D. Appendicitis. Its Surgical Treatment ... 505

PAGE

Crutcher, Howard, M. D. Circumcision Not a Minor Operation..... 235
Hahnemann's Influence Upon Surgery..... 401
Curantur *vs.* Curentur..... 532
Cured Symptoms. C. M. Boger, M. D. 371, 471

Dahlke, Dr. Asthma from a Suppressed Eruption..... 234
Delicate, Backward, Puny, and Stunted Children. By J. Compton Burnett, M. D. Review of... 55
Deschere, Dr. Martin..... 255
Diabetes..... 245
Diagnosis and Treatment of Diseases of the Rectum and Anus. By S. G. Gant, M. D. Review of..... 539
Dietrick, P., M. D. Provings..... 525
Diphtheria and Anti-toxin..... 41
Diphtheria and the So-called Anti toxine. Jos. Fitz Mathew, M. D. 100, 238
Diphtheria Statistics..... 50
Diseases of Rectum and Anus. Review of..... 54
Diseases of the Respiratory Passage. By Charles Porter, M. D. Review of..... 102
Donts for Consumptives. Dr. Charles Wilson Ingham. Review of..... 105
Drake, Olin M., M. D. Warts and Figwarts..... 347
Dudley, Pemberton, M. D. The Centennial Addresses on the Law of Similars..... 188
Dynamics of Natural and Homœopathic Science. B. Fincke, M. D. 256

Eaton, Samuel L., M. D. New Sanatorium..... 252
Eczema of Ten Years' Duration Cured with Mercurius-sol-h.[5m]. E. V. Ross, M. D..... 484
Editorials, 1, 61, 109, 157, 205, 253, 301, 339, 373, 421, 455, 493
Electrolysis for the Surgical Treatment of Strictures..... 57
Electro-therapeutists, Proceedings of the Society of..... 12
Enos, Clinton, M. D. Antimonium Tartaricum..... 22
Euthanasia Under Homœopathy. Wm. Keaney, M. D..... 165
Exanthema Squamosum from Quinine..... 234

Fahnestock, Dr. C. S..... 154
Ferson, Dr. John L. In Memoriam..... 333
Fever Remedies, A Study of. E. W. Boardman..... 329
Fincke, B., M. D. Dynamics of Natural and Homœopathic Science... 256
A Milwaukee Test in Germany..... 159
President's Address..... 424, 458
Fisher, Arthur, M. D. The Story of the Liver..... 79
Fisher, Dr. C. E..... 491
Fisher's Handbook of the Diseases of Children. Review of..... 106
Fisher's Handbook on the Diseases of Children. Dr. Montague R. Leverson..... 184
Fitting Facts to Theories..... 50
Fitz-Mathew, Jos., M. D. Diphtheria and the So-called Anti-toxine... 100, 238

PAGE

Fitz-Mathew, Jos., M. D. Lachesis to the Rescue..... 30
Fœtus in Abdomen of an Adolescent of the Masculine Sex..... 293
Formica-rufa. J. W. Thomson, M. D... 32
Foster, William Davis, M. D. Hernia—Surgical Treatment and Radical Cure..... 322
Fought for Triplets..... 492
Friendship of Dr. Raue and Dr. Hering, C. B. Knerr..... 472
Fun for Doctors..... 60
Fundus Oculi, The. Macmillan & Co.. 337
Funk & Wagnalls. The Standard Dictionary..... 336

Genito-urinary System, Practical Working Handbook of Diseases of..... 490
Goullon, Dr. H. Ammonium-bromatum in Bronchial Troubles..... 244
Grady, Dr. Mary E. In Memoriam..... 334
Great Desideratum, The. E. W. Berridge, M. D..... 449
Guernsey, H. N., M. D. Sea Sickness—Therapeutic Indications..... 243

Hahnemann Association of New York, The..... 40
Hahnemann's Birthday..... 251
Hahnemann Club of Philadelphia, Pa., The..... 58
Hahnemann's Defense of *The Organon*. Review of..... 488
Hahnemann's Influence Upon Surgery. Howard Crutcher, M. D... 401
Hahnemann. Life and Letters of. By Thos. L. Bradford. Review of... 245
Hahnemann Monument, The..... 150
Hahnemannians, The New Society of. 47
Handbook of the Diseases of Children and Their Homœopathic Treatment. Charles E. Fisher, M. D. Review of..... 106
Headache. C. M. Boger, M. D..... 244
Heat Worse by, Better by Wrapping, yet..... 189
Hering on Typhoid Fever.....303, 454
Hering and Raue, Friendship of..... 472
Hernia—Surgical Treatment and Radical Cure. William Davis Foster, M. D..... 322
Hints and Suggestions in the Care and Preservation of the Teeth. By Charles G. Pease, M. D..... 199
Hitchcock, Dr. Charles F..... 154
Holbrook, E. H., M. D. Tallapoosa as a Health Resort..... 371
Homœopathic Medical College of Missouri..... 203
Homœopathic Philosophy. The Study of. W. A. Yingling, M. D..... 189
Homœopathy, The Centennial of..... 147
Homœopathy, Compend of the Principles of. By Wm. Bœricke, M. D. Review of..... 489
Homœopathy and Surgery in Their Relation to Gynecology. Eric Vondergoltz, M. D..... 395
Honor to Our Brother..... 237

Idiosyncrasy and Ipecac. E. V. Ross, M. D..... 484
Imagination in Medicine. Stuart Close, M. D..... 38
Indiana Institute of Homœopathy..... 154

PAGE
Infinitesimals from a Scientific Standpoint. J. M. Selfridge, M. D....... 68
In Memoriam. Dr. John L. Ferson..... 333
In Memoriam. Dr. Mary E. Grady, of Brooklyn....... ......................... 334
In Memoriam Dr. Chas. G. Raue...... 413
International Hahnemannian Association.......................202, 335, 338
International Homœopathic Congress, 151, 250
International Medical Annual and Practitioner's Index.................. 200
Interstate Committee of the American Institute................... ................ 337
Ipecac, Idiosyncrasy and.................. 484

James, Dr. Bushrod W........................ 59
James, Walter M. Arsenicum-album, 1, 61, 109
James, Walter M., M. D. Mercurius-vivus...........157, 205, 253, 301, 339, 373
Nitric acid............... .........421, 455, 493
The Rœntgen Rays......................... 111

Keaney, William, M. D. Euthanasia Under Homœopathy.................... 165
Knerr, Calvin B., M. D. Friendship of Dr. Raue and Dr. Hering....... 472
Kunkel, Dr. Cases........................... 43

Lachesis to the Rescue. Jos. Fitz-Mathew, M. D.......................... 80
Lachesis—Its Origin and Pathogenetic Effects. Prof. W. E. Leonard, M. D........................................ 27
Lee, Edmund J., M. D. Allopathic Progress....................................... 64
Leonard, Prof. W. E. Lachesis—Its Origin and Pathogenetic Effects. 27
Leverson, Montague R., M. D. Dr. Fisher's Handbook on the Diseases of Children........................ 184
Vaccination in the Light of the Royal British Commission.......... 496
Life and Letters of Dr. Samuel Hahnemann. By Thomas Lindsley Bradford, M. D........................... 245
Lutze, F. H., M. D. Sea Sickness...522, 523

Manhattan Life Insurance, New York. 299
Marchand's Glycozone......................... 251
Materia Medica Conference................ 145
Materia Medica, Introductory to the Bureau of. W. A. Yingling, M. D........................................... 374
Maywood Bicycle, The... ............ 299, 454
Measles a Dangerous Disease.............. 241
Medical Century......... ................ 155, 249
Medicine Glass Cover, A..................... 337
Medicine and Surgery in the Twentieth Century. Henry W. Roby, M. D.. .................................... 315
Menninger, C. F., M. D. The Conquest. 304
Mercurius-sol-h.$^{5m}$, Eczema Cured by. 484
Mercurius-vivus. Walter M. James, M. D,......... 157, 205, 253, 301, 339, 373
Metaphysical Magazine, The.............. 360
Milwaukee Test in Germany. A. B. Fincke, M. D............................. 159
Mineral Products of the United States. 419
Modern School of Medicine, The. Dr. Villers.......................................... 126
Morgan, William L., M. D. Vital Nourishment and Similia......... 383
Morphine Symptoms, Confirmation of. Thomas Skinner, M. D........ 123

PAGE
Morphine. See "Vertigo Worse from Motion"........................................ 295
Morphine-Sulphate, Symptoms from. E. V. Ross, M. D........................ 524
Morrow, H. C., M. D. Better by Wrapping, Yet Worse by Heat........... 189
Chionanthus in Jaundice...... ...... 271
Chionanthus-virginica ................. 273
Syphilinum........................................ 212
Syphilinum Cases.................. 129, 209
Syphilinum in Cancerous Ulcerations.................................................. 210
Syphilinum, Comments on.......... 211

National Association of Physicians and Surgeons, The............................ 59
New York Homœopathic Union, Proceedings of the.......................... 366
Nitric-acid. Walter M. James, M. D., 421, 455, 493
Noe, Dr. Amon T........................ 154, 204
Non-Heredity of Inebriety, The. By Leslie E. Keeley, M. D. Review of.................................................. 50
Notes and Notices, 57, 154, 202, 249, 299, 337, 420, 454, 490, 540
Notice.................................................. 540
Nynaism. ...........................................452

*Observer, The*........................... 153, 336, 452
Olds, C. L., M. D. Agnus Castus......... 207
Ailanthus-gland................................. 501
Complementary Remedies............. 290
Conditions Affected by Storms...... 186
Proving of Oleum Animale........... 433
*Organon*, Hahnemann's Defense of the. Review of....................................... 488
*Organon* and Materia Medica Club of the Bay Cities of California, The. 3, 92, 95, 135, 139, 167, 171, 176, 214, 218, 222, 274, 280, 283, 285, 354, 359, 362, 404, 442, 486, 527
Ostrom, Dr. Homer I........................... 154
Our Animal Friends.............................. 454
Outlook, The. W. A. Yingling, M. D.. 437

Pennsylvania State College............252, 299
Perspiration After Sleep. Frederic Preston, M. D.............................. 449
Phenol Dressing.................................. 300
Philadelphia Polyclinic, The............... 411
Pleasures of Out-Door Life.............156, 249
Plumbum Metallicum......................... 154
Pneumatic Truss Pads......................... 300
Practical Working Handbook of Diseases of Genito-urinary System... 490
Practice of Medicine, The. By Marvin A. Custis, M. D............................ 248
Pregnancy, Labor, and the Puerperal State. By Egbert H. Grandin, M. D. Review of........................ 108
President's Address. B. Fincke, M. D. 424, 458
Preston, Frederic, M. D. Perspiration After Sleep.................................. 449
Principles of Surgery. By N. Senn, M. D. Review of...................... 104
Proceedings of the I. H. A., The.......... 420
Proll, Dr. A Remarkable Case........... 232
Proving of Oleum Animale. C. L. Olds, M. D...................................... 433
Provings. P. Dietrick, M. D.............. 525
Psorinum. J. H. Allen, M. D............. 324

Quinine Kills a Child......................... 156

Raue, Dr. Charles G. In Memoriam.. 413

PAGE
Raue and Hering, Friendship of......... 472
Reasonable Suggestion........................ 50
Rectum and Anus, Diseases of. Review of........................................ 539
Remarkable Case, A. Dr. Proll.......... 232
Rœntgen Rays, The. Walter M. James, M. D........................................ 111
Repertory of Hering's Guiding Symptoms of our Mat. Med. By Calvin B. Knerr, M. D. Review of....... 51
Repertory of Tongue Symptoms. By M. E. Douglass, M D. Review of. 489
Repetition of the Dose, The................ 267
Repetition of the Dose. B. L. B. Baylies, M. D........................................ 440
Roby, Henry W., M. D. Medicine and Surgery in the Twentieth Century........................................ 315
Rogers, Dr. L. D............................299, 420
Ross, E. V., M. D. Idiosyncrasy and Ipecac........................................ 484
Eczema of Ten Years' Duration Cured with Merc-sol.................. 484
Symptoms from Morphine-Sulphate........................................ 524

Santonine Poisoning, Case of............... 294
*Scientific American, The*..................419, 492
*Scientific American*, Fiftieth Anniversary Number of........................... 490
Scientific Management of Pulmonary Tuberculosis. By C. W. Ingham, M. D........................................ 295
Sea Sickness—Therapeutic Indications. H. N. Guernsey, M. D...... 243
Sea Sickness. F. H. Lutze, M. D...522, 523
Selfridge, J. M., M. D. Infinitesimals from a Scientific Standpoint....... 68
Self-Protection in Boston..................... 492
Sheffield, Henry, M. D. Amalgam and Kindred Poisons.................. 228
Silicea........................................ 294
Similars, The Centennial Address on the Law of. Pemberton Dudley, M. D........................................ 189
Skinner, Thomas, M. D. Confirmation of Morphine Symptoms............... 123
Smith, C. Carleton, M. D. Conditions Affected by Storms,..................... 101
Society of Electro-therapeutists. See Electro-therapeutists.
Standard Dictionary of Funk & Wagnalls........53, 155, 249, 336, 454, 490, 491
Stibrium Arsenicosum........................ 154
Storms, Conditions Affected by. C. L. Olds, M. D.................................. 186
Story of the Liver by Andrew Wilson, The. Arthur Fisher, M. D......... 79
Strictures, Electrolysis for the Surgical Treatment of.............................. 57
Surgery, Principles of. By N. Senn, M. D. Review of......................... 104
Surgery Without Pain......................... 240
Surgical Cleanliness. Dr. L. D. Rogers. Review of.................................. 106
Symptom Covering............................ 408

PAGE
Symptoms from Morphine-Sulphate. E. V. Ross, M. D......................... 524
Syphilinum. H. C. Morrow, M. D...... 212
Syphilinum in Cancerous Ulcerations. H. C. Morrow, M. D.................... 210
Syphilinum Cases, Some. H. C. Morrow, M. D. ...........................129, 209
Syphilis in the Middle Ages and in Modern Times. By Dr. F. Buret. 296

Tallapoosa as a Health Resort. E. H. Holbrook, M. D............................ 371
Teeth, Hints and Suggestions in the Care and Preservation of the. Review of........................................ 199
Therapeutical Applications of Peroxide of Hydrogen. By Charles Marchand........................................ 418
Thomson, J. W., M. D. Formica-rufa.. 32
Tobacco, A Rare Effect of................... 420
Tongue Symptoms, Repertory of. By M. E. Douglass, M. D Review of. 489
Transactions of the Fifty-first Session of the American Institute of Homœopathy.............................. 153
Transactions of the Thirty-first Session of the Homœopathic Society of Pennsylvania.......................... 202
Truth Shall Make us Free, The. J. H. Allen, M. D......................18, 162, 351

Urinalysis. Joseph C. Guernsey, M. D. Review of.................................... 54

Vaccination in the Light of the Royal British Commission. M. R. Leverson, M. D........................................ 496
Vaccination, No Marriage Without..... 420
Vertigo Worse from Motion. C. M. Boger, M. D................................. 295
Villers, Dr. The Modern School of Medicine........................................ 126
Vital Nourishment and Similia. William L. Morgan, M. D..................... 383
Vomiting of Fluids but Not of Solids. W. A. Yingling, M. D..................... 48
Vondergoltz, Eric, M. D. Homœopathy and Surgery in Their Relation to Gynecology................... 394
Voter's Guide, The.............................. 453

Warts and Figwarts. Olin M. Drake, M. D........................................ 347
Waterhouse, Amelia A., M. D. The Cause........................................ 39
Weir's Index to the Medical Press....... 201
Wrapping, Better by........................... 189

Yingling, W. A., M. D. Introductory to the Bureau of Materia Medica........................................ 374
The Outlook.................................. 437
The Study of Homœopathic Philosophy........................................ 189
Vomiting of Fluids but Not of Solids........................................ 48

VOL. XVI. JANUARY, 1896. No. 1.

### THE HOMŒOPATHIC PHYSICIAN,

A Monthly Journal of Homœopathic Materia Medica and Clinical Medicine.

*"He is a freeman whom truth makes free, and all are slaves beside."*

*"Seek the Truth: come whence it may, cost what it will."*

EDITED AND PUBLISHED BY

WALTER M. JAMES, M. D.

NOTICE.

***All articles for publication, books for review, business communications, etc., must be sent to Editor, 1231 Locust Street, Philadelphia.***

***Subscribers will please notice that this Journal will be sent until arrears are paid and it is ordered discontinued.***

PHILADELPHIA:

